# Continuing Professional Development via Social Media or Conference Attendance: A Cost Analysis

**DOI:** 10.2196/mededu.6357

**Published:** 2017-03-30

**Authors:** Stephen Maloney, Jacqueline Tunnecliff, Prue Morgan, James Gaida, Jennifer Keating, Lyn Clearihan, Sivalal Sadasivan, Shankar Ganesh, Patitapaban Mohanty, John Weiner, George Rivers, Dragan Ilic

**Affiliations:** ^1^ Department of Physiotherapy. Monash University Frankston Australia; ^2^ Epworth Health Care Melbourne Australia; ^3^ University of Canberra Research Institute for Sport and Exercise (UC-RISE) Canberra Australia; ^4^ Monash University Melbourne Australia; ^5^ Monash University Sunway Malaysia; ^6^ Composite Regional Center for Persons with Disabilities Lucknow India; ^7^ Swami Vivekanand National Institute of Rehabilitation Training and Research Cuttack India

**Keywords:** social media, knowledge translation, continuing medical education

## Abstract

**Background:**

Professional development is essential in the health disciplines. Knowing the cost and value of educational approaches informs decisions and choices about learning and teaching practices.

**Objective:**

The primary aim of this study was to conduct a cost analysis of participation in continuing professional development via social media compared with live conference attendance.

**Methods:**

Clinicians interested in musculoskeletal care were invited to participate in the study activities. Quantitative data were obtained from an anonymous electronic questionnaire.

**Results:**

Of the 272 individuals invited to contribute data to this study, 150 clinicians predominantly from Australia, United States, United Kingdom, India, and Malaysia completed the outcome measures. Half of the respondents (78/150, 52.0%) believed that they would learn more with the live conference format. The median perceived participation costs for the live conference format was Aus $1596 (interquartile range, IQR 172.50-2852.00). The perceived cost of participation for equivalent content delivered via social media was Aus $15 (IQR 0.00-58.50). The majority of the clinicians (114/146, 78.1%, missing data n=4) indicated that they would pay for a subscription-based service, delivered by social media, to the median value of Aus $59.50.

**Conclusions:**

Social media platforms are evolving into an acceptable and financially sustainable medium for the continued professional development of health professionals. When factoring in the reduced costs of participation and the reduced loss of employable hours from the perspective of the health service, professional development via social media has unique strengths that challenge the traditional live conference delivery format.

## Introduction

Professional development is essential in the health disciplines. It allows the health workforce to maintain clinical currency, informed by emerging evidence, and supports best practice [1,2]. Best practice is continually changing and requires clinicians, researchers, and educators to be lifelong learners [3]. Certifying a minimum number of professional development hours is a requirement across many health disciplines for maintenance of ongoing registration to practice and to maintain best practice [4]. Together, these development activities support risk management and quality assurance activities within health care services [5,6].

Continuing professional development (CPD) can take many forms—from in-services and journal clubs, to courses and conferences, and Web-based activities. Each mode of engagement has its own unique strengths and weaknesses [7]. A journal club allows intimate discussion of a clinical issue, however it may be limited by the clinicians’ abilities to critique and contextualize the evidence presented [8]. A conference provides access to breaking evidence from around the globe, supplemented by expert critiques; however, the time and financial costs of attendance may be prohibitive [7]. Web-based activities may provide cheaper avenues to emerging information and extend geographical and time-boundaries; however, limitations also exist with verifying the quality and credibility of the information source. Web-based mediums provide a nontraditional mode of social and professional engagement, with its own strengths and weaknesses based on the individual’s perspective—connecting profiles and facilitating asynchronous conversation and information exchange [9], but foregoing the benefits of authentic live dialogue [10].

The majority of clinically relevant evidence does not survive the journey from researchers to clinicians at the point of care [3]. This remains true despite the significant resources being allocated to CPD and education. The evidence-to-practice gap is magnified by time pressures on clinicians, difficulties in searching and accessing the evidence, the challenge of assessing whether the evidence is applicable to a particular patient, and having the knowledge, skills, and resources to realize when and how to act upon that evidence [11].

Social media is designed to level information hierarchies, allowing the user to contribute directly to the sharing of information between individuals and communities of practice. Social media is now a mainstream information-sharing pathway, in part due to its speed, worldwide reach, and flexibility in access [12]. Maloney et al investigated the impact of social media on learning and translation to practice within the health disciplines [13]. This pre-post study of 199 clinicians across 4 continents provided practice points on evidenced-based tendon management, which were delivered only though social media. After the study, clinicians had more positive perceptions of social media for professional development, demonstrated an improvement in content knowledge, and reported intended management of patients that more closely aligned with current evidence [13]. Of all the participants, 80% (120/150) believed that social media would play a very significant role in the translation of evidence to practice. However, the participants also expressed caution in adopting a social media–led CPD environment, based primarily on concerns of trustworthiness of the evidence presented. These are valid concerns, as social media does not have the quality control mechanism of peer review. The strength of many social media platforms is that they are free and have open access, with the potential to reduce the cost of translating knowledge from researchers to clinicians.

Knowing the cost and value of educational approaches informs decisions and choices about learning and teaching practices [14]. It informs the sustainability of teaching approaches, and the efficiency and reach of workforce training development [15]. This is particularly relevant in times of health workforce shortages in rural and remote settings, and in times of budget restrictions within health services and education institutions. Despite the push for increased fiscal responsibility and accountability in health professional education, economic evaluations of cost and value remain uncommon [14-16].

Analyses of costs involved in CPD of clinicians can be conducted from many viewpoints. The provider incurs costs of delivering the content, participation costs are borne by the learner, and the health service has significant costs in the release and subsidy of staff to attend professional development activities [15]. Where health services are publicly funded, the cost is also borne by the taxpayer. Conferences have the largest opportunity cost for all stakeholders, primarily due to reduced capacity for health service provision with clinical staff attendance. There is currently no literature investigating the cost of participation in CPD delivered through social media to health professionals.

The primary aim of this component of the investigation was to analyze the opportunity costs of participation in CPD of the health disciplines via social media, compared with live conference attendance. The secondary aim was to investigate the acceptability of social media as a method for translating evidence to practice for clinicians, from the perspective of willingness-to-pay analysis of health professionals for a hypothetical social media professional development subscription.

## Methods

### Design

A cross-sectional design was undertaken to answer the research questions. Quantitative data were obtained from an anonymous electronic questionnaire. Ethics approval was obtained through the Monash University Human Research Ethics Committee (Approval number CF14/1372-2014000640).

### Participants

This study was completed as one arm of a larger study investigating the potential role of social media for CPD and translational research. The detailed methods of this study are provided elsewhere [13]. In brief, this arm of the study invited participation from clinicians who were interested in musculoskeletal care. The invitation to participate was distributed via social media, as well as via email to the clinical affiliations of Monash University, Faculty of Medicine, Nursing and Health Sciences, Australia; Monash University Malaysia; Warwick Medical School, University of Warwick, United Kingdom; and Swami Vivekanand National Institute of Rehabilitation Training and Research, India. The email contained a hyperlink for participants to accept the invitation to take part in the survey, as well as a link to decline to participate. If participants chose to decline, an option was available for them to volunteer their reason for not participating.

Of the clinicians, educators, and researchers who accepted the invitation, a filter was applied to reduce the potential participants to only those clinicians interested in musculoskeletal care (n=272), thereby aligning their field of interest with the scenarios presented in the study. No restrictions were made to the health discipline, age, or country of the participants. Undergraduate students were eligible to participate if they were actively engaged in the clinical practice phase of their education.

### Outcomes

In the absence of a validated survey to obtain the required financial data for this population and context, a survey was developed by the research team including a tool to elicit willingness to pay for particular services using a stated preference approach [15]. The survey questions relating to the economic analyses are provided in Multimedia Appendix 1. The survey was designed to elicit the following themes:

1. Demographic data regarding age, country, and health discipline.
2. Perception of educational outcomes contrasting live conference attendance with social media–delivered professional development.
3. Participants’ willingness to pay for any of the three options: a live conference format, social media–based format, and a live conference supplemented by a social media platform.
4. Participants’ perceived costs of participation in live conference attendance, compared with a social media–based platform for the same information content.
5. The perceived number of effective employment work-hours lost through attending a live conference format, compared with a social media–based format.
6. Participants’ willingness to pay for a hypothetical social media subscription that provides evidence and practice updates.

The “conference” scenario provided to contextualize the question to participants within the survey was as follows:

There is a conference being offered in the field of your area of clinical interest. It has leading local and international experts in the field to present their latest research, and discussions and panels on key issues in the field. The conference is two days duration, held on a Wednesday and Thursday, in Melbourne, Australia.

Live conference attendance costs included conference fees, travel, accommodation, meals, and any other significant expenses anticipated by the respondent. Costs for attendance via social media included Internet access costs, along with any other significant costs anticipated by the respondent. A generic attendance fee price was set by first converting the respondent’s answers to their willingness to pay to the same currency (Australian dollars) and then calculating the willingness to pay for each format.

**Figure 1:**
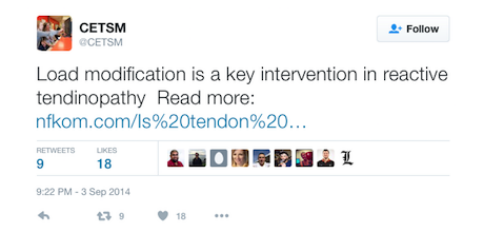
A conceptual example of a Twitter-based short course for musculoskeletal practice.

The hypothetical subscription service was described to participants as being able to deliver 1 message per week of 150 characters, concerning updates in practice in the field of musculoskeletal care, for 48 weeks of the year, with a link to further source of information such as a journal article. To help contextualize a social media post of this nature, a screen-image of an example is provided in Figure 1 taken from the Twitter-based short course within another arm of this research [13].

All financial data contributed were converted to Australian dollars before computations were made. Currency conversion rates were taken from Xe.com, on October 20, 2014. Data were presented in summary format as median and IQR in Australian dollars. The average work-day length was taken at 7.25 hours.

**Table 1:** Demographic data of the participants.

Descriptor		Participants n (%)	
**Country**			
	Australia		59(39)
	United Kingdom		33 (22)
	India		12 (8)
	United States		17 (11)
	Malaysia		5 (3)
	Other^a^		24 (16)
**Age (in years)**			
	18-24		34 (22)
	25-34		68 (45)
	35-44		34 (22)
	45-54		10 (6)
	>54		4 (2)
**Profession**			
	Physiotherapy		117 (78.0)
	Medicine		19 (12)
	Osteopathy		3 (2)
	Podiatry		9 (6)
	Other		2 (1)

^a^The country category “other” is made up of any country that had less than three participants.

## Results

### Demographics

Of the 272 clinicians invited to contribute data to this study, 150 completed the study’s outcome measures (150/272, 55.1%). Participant demographic details are represented in Table 1.

### Subscription Value

The majority (114/146, 78.1%, missing data n=4) of respondents indicated that they were willing to pay for a social media subscription service targeting their area of professional development need. The hypothetical service delivered was 1 message per week of 150 characters, concerning updates in practice in the field of musculoskeletal care, for 48 weeks of the year, with a link to further source of information such as a journal article. The median price, reported for those who indicated they were willing to pay for a subscription, was Aus $59.50 (IQR 29.75-106.25, missing data n=18).

### Willingness to Pay: Live Conference Versus Social Media

One-third of the respondents (50/150, 33%) felt that they would have the same educational outcomes whether the information was delivered within a live conference format, or via a social media–based format. Half of the respondents (78/150, 52%) believed that they would learn more with the live conference format, and the remaining 14% (22/150) felt that they would learn more from the social media–based format.

The respondents reported a willingness to pay for live conference registration in the order of Aus $342 (IQR 171.00-500.00, missing data n=18). Their willingness to pay for the social media–based equivalent was valued 68% less, at Aus $110.50 (IQR 50.00-200.00, missing data n=20). The live conference format supplemented by a social media platform was valued at an equivalent rate to the live conference only, at Aus $350.00 (IQR 156.00-500.00, missing data n=19).

The median perceived participation costs for the live conference format (not including registration) was Aus $1596 (IQR 172.50-2852.00, missing data n=19). The cost of participation for the social media–based equivalent is Aus $15 (IQR 0.00-58.50, missing data n=31). A subanalysis looking at Australian-based clinicians who would not need international travel to attend the base scenario conference (n=54), placed the median cost of participation for live conference attendance at Aus $122.50 (IQR 50.00-506.25).

### Service Delivery Hours

Respondents indicated that they would need to access a median of 2.76 days of leave (20.00 hours, IQR 16.00-37.50, missing data n=17) to attend the mock conference presented in the base scenario. In contrast, the respondents reported that they would require just over one day of leave (7.75 hours, IQR 0.00-15.00, missing data n=26) to attend the social media–based equivalent.

## Discussion

### Principal Findings

The perceived costs of participation in CPD via social media, compared with live conference attendance, were far lower for the social media–based equivalent format, regardless of whether travel costs were included or excluded. Findings suggest that the potential savings in staff hours for a health service provider, releasing staff from clinical duties for attendance of CPD activities, were 12.25 hours per attendee. Interestingly, approximately half (72/150, 48%) of the respondents indicated that they believed they would learn either the same, or more, from the social media–based format than its live conference alternative.

Participants rated their willingness to pay for the social media–based format substantially lower than the live face-to-face alternative, indicating that it is of a lower perceived value. This may reflect the perception of lower costs to the education provider, which should be passed on to the consumer as a reduced registration fee [17]. This could be an accurate assumption by the respondents, given that one of the unique features of any education delivered by social media is its scalability. Unlike a live conference delivery format, social media incurs minimal variable costs with increased numbers of participation [15].

The majority of the clinicians indicated that they would pay for a subscription-based service, delivered by social media, to the value of Aus $59.50. This finding may indicate that there is an increasing readiness in the health professional sector for professional development delivered by social media, or perhaps opportunity for a sustainable business model for providing this service. However, an analysis of the barriers, risks, and rewards for such an activity was outside the scope of this study.

The overarching picture created by this research is not that social media provides equivalent educational benefit to conference presentations for less cost. There are a large number of tangible and intangible benefits to face-to-face conferences, such as networking opportunities, lengthy discussions, along with relaxation and creativity that comes from a change of work environment. Rather, this research provides important first steps scrutinizing the cost-effectiveness of our CPD activities, and the current degree of acceptability of social media as a medium for professional development.

Just as the methods of education are changing for undergraduate education, through simulation, social media, and other technology-enabled pedagogy, so it is in the professional development environment. It is interesting to consider that although didactic lectures are arguably the mode of delivery least supported by evidence to change practice and to generate discussion [18,19], when attending a conference on groundbreaking educational research, a didactic presentation is the most common format provided to the conference presenters. However, it may be that a change is looming in the conference-based learning environment also, with organizers beginning to harness the positives of live attendance, in conjunction with the benefits available from social media. An example is the Medicine 2.0 conference, the world congress of social media, mobile apps in health care and medicine, which was held in Maui, HI in 2014 [20]. This is a progressive conference, which encourages the use of social media by the audience, expanding the presenter’s reach to interested observers, and facilitating discussion about the emerging evidence being presented. Socialization and networking is facilitated through QR codes available on each delegate’s name tag. Scanning the tag of a new contact automatically links the two parties’ social media profiles with the aim of fostering future collaborations. It is feasible that in the near future, a careful design of social media targeted to health professional education will be an acceptable alternative to live conference attendance, rather than simply being an important supplement to the conference’s activities.

### Limitations

There are a number of limitations that may affect the accuracy and generalizability of this study’s findings. The mode of circulating the invitation to participate in this study included social media—and therefore could have created a selection bias toward participants who value social media more highly. The participants were spread across a wide geographic area, which may hide in-country differences; however, this is arguably quite authentic for any CPD provided via social media due to its ability to overcome geographic boundaries. Few who declined the invitation to participate in the study volunteered their reason for doing so, removing the ability to learn more about the nonparticipators and their potential impact on any selection bias. Another limitation is the narrow scope of the study, as it is focused only on the perceived economic costs of the different conference formats. As raised earlier, there are a number of personal or professional factors that may contribute to the participant’s overall perceived benefit and final determination of value for the learning. Perhaps individual learning styles or confidence in navigating and engaging with Web-based technologies affect the feasibility of using social media for professional development. The same could be said for the perspective of the health service, with indirect benefits being obtained such as improved institutional profile. The findings are also influenced by who bears the cost for the education. Some learners would attend conferences through project funds, subsidized by their employers, or through their own salaries, with each variation potentially influencing the consideration of learners to attend CPD activities and the subsequent costs of participation. Likewise, the study did create the assumption that the individual, rather than their workplace, would provide the costs of accessing any social media–based education. As with all stated preference techniques used to elicit willingness to pay, there may be warm glow and part-whole [21] biases, leading to overestimation effects. With respect to warm glow bias, where an individual overestimates their values due to the satisfaction with the act of giving, it is unknown whether the respondents considered their willingness to pay as a form of obligatory compliance to society, as reflected by regulatory requirements for registration, rather than their personal development. Moreover, with respect to part-whole bias, it is unknown whether respondents see their willingness to pay as encapsulating the importance of continuous professional development in their own profession or the importance of CPD across all professions. Although the stated preference approach is commonplace, no validated questions existed to apply the approach specifically to the scenarios of this study—therefore, a customized survey was created.

### Future Work

Further studies within this line of research may choose to evaluate the cost-benefit of a larger variety of CPD methods, with provision for measuring actual knowledge acquisition. The assumption of greater networking and professional socialization occurring via live event attendance in contrast to a purely Web-based format could also be revealing. Given that emerging research is showing evidence of social media being able to positively impact behavior change in the health disciplines [13,22], an alternate research direction could be to focus in on social media only, determining the value of different functional elements of the social media platform, and how the medium could be maximized for learning in the health disciplines or best integrated into traditional conference settings. Larger studies on this topic may also be well placed to investigate in-country and between-country differences, such as the influence of different pay structures and its effect on determining value.

### Conclusions

Social media platforms are evolving into an acceptable and sustainable medium for the continued professional development of health professionals. In contrasting a 2-day live conference under the conditions of this study to equivalent content via social media, approximately half of the clinicians felt that they would learn the same, or more, via the social media–based format. When factoring in the significantly reduced costs of participation and the reduced loss of employable hours from the perspective of the health service, professional development via social media has unique strengths that may challenge the traditional live conference delivery format. Further evidence of the increasing role of social media in the translation of emerging evidence to clinical practice is highlighted in 78.1% (114/146) of the clinicians indicating their willingness to pay for a social media subscription, which would provide a weekly evidence-based practice point in their field of clinical interest. It is anticipated that professional development via social media will continue to offer viable and more cost-effective options than the more traditional methods currently available. Further investigations into CPD that include considerations of cost and value are important for ongoing improvement in the effectiveness and efficiency of our health workforce skills and training.

## Appendix

Multimedia Appendix 1 Questionnaire.

## Abbreviations

CPD: continuing professional development
IQR: interquartile range

## References

[1] Shepard K, Jensen G (2002). Handbook of teaching for physical therapists. 2nd Edition.

[2] Whitcomb ME (2002). CME reform: an imperative for improving the quality of medical care. Acad Med.

[3] Glasziou P, Haynes B (2005). The paths from research to improved health outcomes. ACP J Club.

[4] (2014). AHPRA.

[5] Physiotherapy Board of Australia (2015). Guidelines: Continuing Professional Development. Australian Health Practitioner Regulation Agency.

[6] Foo JS, Storr M, Maloney S (2016). Registration factors that limit international mobility of people holding physiotherapy qualifications: a systematic review. Health Policy.

[7] Davis D, O'Brien MA, Freemantle N, Wolf FM, Mazmanian P, Taylor-Vaisey A (1999). Impact of formal continuing medical education: do conferences, workshops, rounds, and other traditional continuing education activities change physician behavior or health care outcomes?. J Am Med Assoc.

[8] Straus S (2011). Evidence-based medicine: how to practice and teach it.

[9] (2016). press.LinkedIn.

[10] Tunnecliff J, Ilic D, Morgan P, Keating J, Gaida JE, Clearihan L, Sadasivan S, Davies D, Ganesh S, Mohanty P, Weiner J, Reynolds J, Maloney S (2015). The acceptability among health researchers and clinicians of social media to translate research evidence to clinical practice: mixed-methods survey and interview study. J Med Internet Res.

[11] Ilic D (2009). Assessing competency in evidence based practice: strengths and limitations of current tools in practice. BMC Med Educ.

[12] Maloney S, Moss A, Ilic D (2014). Social media in health professional education: a student perspective on user levels and prospective applications. Adv Health Sci Educ Theory Pract.

[13] Maloney S, Tunnecliff J, Morgan P, Gaida JE, Clearihan L, Sadasivan S, Davies D, Ganesh S, Mohanty P, Weiner J, Reynolds J, Ilic D (2015). Translating evidence into practice via social media: a mixed-methods study. J Med Internet Res.

[14] Walsh K, Reeves S, Maloney S (2014). Exploring issues of cost and value in professional and interprofessional education. J Interprof Care.

[15] Maloney S, Haas R, Keating JL, Molloy E, Jolly B, Sims J, Morgan P, Haines T (2012). Breakeven, cost benefit, cost effectiveness, and willingness to pay for web-based versus face-to-face education delivery for health professionals. J Med Internet Res.

[16] Maloney S (2014). Twitter.

[17] Haines TP, McPhail S (2011). Patient preference for falls prevention in hospitals revealed through willingness-to-pay, contingent valuation survey. J Eval Clin Pract.

[18] Ilic D, Maloney S (2014). Methods of teaching medical trainees evidence-based medicine: a systematic review. Med Educ.

[19] Roche A, Eccleston P, Sanson-Fisher R (1996). Teaching smoking cessation skills to senior medical students: a block-randomized controlled trial of four different approaches. Prev Med.

[20] (2014). Medicine20congress.

[21] Robertson LC, Lamb MR (1991). Neuropsychological contributions to theories of part/whole organization. Cogn Psychol.

[22] Tunnecliff J, Weiner J, Gaida JE, Keating JL, Morgan P, Ilic D, Clearihan L, Davies D, Sadasivan S, Mohanty P, Ganesh S, Reynolds J, Maloney S (2016). Translating evidence to practice in the health professions: a randomized trial of Twitter vs Facebook. J Am Med Inform Assoc.

