# Supplementary material for: Continuing Professional Development via Social Media or Conference Attendance: A Cost Analysis
Source: JMIR Med Educ. 2017 Mar 30;3(1):e5. doi: 10.2196/mededu.6357 (PMC5391434; doi:10.2196/mededu.6357)
Supplement: Multimedia Appendix 1 [file mededu_v3i1e5_app1.pdf]

Appendix 1: Questionnaire

Page Introduction:

We are interested in your thoughts and opinions related to cost and value when using social media for professional purposes

1. Consider the following scenario.

There is a conference being offered in the field of your area of clinical interest. It has leading local and international experts in the field to present their latest research, and discussions and panels on key issues in the field. The conference is two days duration, held on a Wednesday and Thursday.What do you think would be a fair registration price (assuming no membership or discount) for the described conference if:

- It is only delivered face-to-face? (Please specify amount and currency)
- It is delivered face-to-face, but is supported by a social media allowing delegates to discuss highlights, and have presenters involved in the discussions? (Please specify amount and currency)
- If it was delivered via social media only (password protected), with no face-to-face presentations, instead having video presentations, and then the presenter available for discussion/questions, and the discussion can continue between delegates? (Please specify amount and currency)

2. If the conference in the above scenario was to be held in Melbourne city, Australia, what would be your estimated total cost for attending in person? You should consider transport costs from your residence, accommodation costs, meals and any other costs you would anticipate occurring (excluding registration fees). Please specify the amount and currency.

- Transport
- Accommodation
- Meals and other costs

3. Everyone has different habits at conferences, for example, how many sessions they attend, how much they listen, how much they network. You completed a 'test on your tendon knowledge' earlier in this survey. Do you think you would have gone better, worse, or the same if the same information had been given at a conference, rather than by social media?

- I would have done better
- I would have done the same
- I would have done worse
- Why? (please explain your above selection).

4. If the conference in the above scenario was to be held in Melbourne city, Australia, what would be your estimated total costs of participation if you were to participate via social media only? You should consider internet costs on the device you would most likely use (i.e. phone or

laptop) and any other costs you would anticipate occuring. Please specify amount and currency.

5. How many working hours would you need to take off for the conference in the above scenario if you were to:

- Attend the face-to-face conference scenario?
- Participate via social media only?

6. Would you consider paying a subscription to ‘practice points’ delivered via social media, such as those that you experienced in this study, created and sent by a leading researcher in your field of interest, keeping you up to date with research and practice advancements and events (1 message of 150 characters each week, for 48 weeks of the year, with a link to a full source of information, such as a journal article)?

- Yes
- No

7. If you answered yes to the above question, how much would you pay for an annual subscription? Please specify amount and currency.
